# Contraceptive Use and the Associated Factors among Women of Reproductive Age in Jazan City, Saudi Arabia: A Cross-Sectional Survey

**DOI:** 10.3390/ijerph20010843

**Published:** 2023-01-02

**Authors:** Mohamed Salih Mahfouz, Mona Elmahdy, Majed Ahmed Ryani, Amani Osman Abdelmola, Samah Ahmed Ali Kariri, Hayat Yahya Ahmad Alhazmi, Salwa Hussain Mater Almalki, Ons Mohammed Adhabi, Sahar Mohammed Ali Hindi, Nouf Mousa Muqri, Bashayer Abdullah Towhary

**Affiliations:** 1 Department of Family and Community Medicine, Faculty of Medicine, Jazan University, Jazan 45142, Saudi Arabia; 2 Faculty of Medicine, Benha University, Benha 13511, Egypt; 3 Faculty of Medicine, Jazan University, Jazan 45142, Saudi Arabia

**Keywords:** contraceptive methods, reproduction, pregnancies and Jazan

## Abstract

The contraceptive use profile is poorly understood in some Gulf Arabian countries, including Saudi Arabia. The present study aimed to assess the knowledge, attitude, and practices related to contraceptive use among women of childbearing age in Jazan city, Saudi Arabia. An observational, analytical cross-sectional study was conducted among a random sample of 450 women in Jazan city. The data were obtained through personal interviews using a questionnaire that included questions on women’s level of knowledge about contraception methods, their attitudes towards contraception methods, and their practices. Data were analyzed using descriptive and inferential statistics techniques using the SPSS program. The prevalence of ever having used contraceptives was significantly different according to age group, job status, children ever born, and the number of pregnancies (*p* < 0.05 for all). On the other hand, the prevalence of desire to use the contraceptives in the future was high, at 70.9%, with a 95% CI [66.5–74.9]. It differed significantly according to age group and job status (*p* < 0.05 for both). The most familiar and usable types of contraceptives were pills (36.3%) and intrauterine devices (24.4%). When asked their reasons for using contraceptives, 53.8% of participants cited child spacing and 21.8% improving child health. Logistic regression revealed that the use of contraception among women was more associated with the 20–34 age group [COR = 7.7, 95% CI = 4.4–13.5, *p* < 0.001] than the 15–24 age group. Having more than one pregnancy and having more than one child were also associated with increased use of contraceptive methods (*p *< 0.001 for both). These results indicate there is a high level of awareness about contraceptives, a positive attitude toward them, and good practices for the use of them among Saudi women in Jazan. More efforts are needed to improve women’s awareness for better utilization of the available services.

## 1. Introduction

Family planning enables couples to obtain their desired number of children and determine the spacing of pregnancies, which is achieved mainly through contraceptive methods [1]. Contraception (birth control) prevents unwanted pregnancy by interfering with the normal process of ovulation, fertilization, and implantation. Birth control methods are numerous and characterized by different mechanisms of action and effectiveness in preventing pregnancy [2]. According to the World Health Organization (WHO), ensuring access to preferred contraceptive methods for women is essential to supporting the health of mothers and children and the community’s economic situation [3,4].

It is well documented that women’s reproductive rights, including the right to decide the number, timing, and spacing of their children, are fundamental to their empowerment and equality [3,4]. The postpartum period is important for initiating contraception, as ovulation can occur as early as 25 days. Contraception use in the extended postpartum period, i.e., one year after the child’s birth, is highly recommended, as the delay of use until the return of menstruation might subject women to the risk of unwanted pregnancy [5].

In 2019, of the 900 million girls and women aged 15–49 years worldwide who were using contraception, 842 million used modern contraception, while 80 million were users of traditional methods [6]. According to the UN, the prevalence of contraceptive use (any method) in Western Asia was 35.3%, while the estimated contraceptive use for the Kingdom of Saudi Arabia (KSA) was 18.6% [6].

The profile of contraceptive use in Saudi Arabia, based on the Saudi Household Survey 2018, indicated that 30.4% of women of reproductive age used contraceptives [7]. Oral contraceptives were the most common method used by Saudi women [8,9,10]. A recent study conducted in Eastern Province, Saudi Arabia, reported the prevalence of unplanned pregnancies as 26.4% [11]. The main determinants of use among Saudi women included age, children ever born, educational level, and family size [12,13], while the main barriers were cultural factors and the belief that contraceptive methods can harm women’s health [8].

In Middle Eastern countries, cultural values and traditions are among the factors that affect the extent of contraception use [14]. Family planning knowledge and use are key factors for health care planning and population policy design. The scope of family planning in southwest Saudi Arabia is not well understood. In this work, we hope to sketch the profile of family planning in Jazan, Saudi Arabia and provide baseline data for further study in developing appropriate evidence-based strategies to promote the use of contraceptives in Jazan. Few studies dealing with contraceptives have been conducted in the Jazan region. Thus, the present study aimed to examine the knowledge and attitudes about and practice of contraception among women of childbearing age in Jazan, and to explore the possible associated factors.

## 2. Materials and Methods

### 2.1. Design, Population, and Setting

This analytical cross-sectional study was conducted in the city of Jazan between April 2018 and May 2018. Its aim was to assess the knowledge, attitudes, and practices related to contraception among women of childbearing age. The eligibility criteria of the study were: age 15–49, attendance at one of five Primary Health Care Centers in Jizan (PHCCs), and willingness to participate in the survey. Jazan is a port city and the capital of the Jazan Region, with a population of 134,764 residents. The region is located in the southwest corner of Saudi Arabia and extends along the Red Sea. It is 13,000 km^2^ in size, and is divided into 16 governorates.

### 2.2. Sampling Procedure

A sample size of 480 women was estimated for this study. We used Cochran’s formula for calculating sample size when the population is infinite. The formula was developed to calculate a representative sample for proportions and is written as follows: [*n* = *Z^2^*_(1−α/2)_ × *P*(*1 − P*)/*d*^2^]. The calculation of the sample size was based on a 95% confidence level with ±5% precision and an anticipated population proportion (P) of 50%. This was the safest choice for (P), as the sample size is largest when P = 50%. We assumed a non-response rate of 20%, so we increased the final sample size by 80 participants. The sample was distributed equally among the five randomly selected PHCCs forming Jazan city. Finally, women were selected from each PHCC using convenience sampling.

### 2.3. Data Collection, Tool, and Piloting

Data were collected by the study team using face-to-face interviews. A standardized questionnaire was used for data collection. The questionnaire was developed by the study team after they consulted the literature in the same field [12,13,15,16]. The final version of the questionnaire had four parts: personal characteristics such as age and education level (illiterate, educated, and well educated); occupational status (housewife, student, and worker); marital status; number of pregnancies; and number of children. The second section of the questionnaire contained questions on women’s knowledge about contraception, the most familiar contraceptive method among participants, their source of knowledge, and their awareness of complications. The third part examined women’s attitudes toward contraceptive use, while the fourth section discussed that use. Questions in the knowledge and practices sections were multiple choice and dictums (yes and no responses). Attitudes toward the use of contraceptive methods was measured using a 5-point Likert scale. A pilot study was conducted among 25 women to test the wording of the questionnaire in order to avoid inter-observer variation or bias. The validity of the questionnaire was determined by the content validity method, and the reliability of the questionnaire was confirmed by determining Cronbach’s alpha coefficient. Cronbach’s alpha coefficient was calculated as 0.760.

### 2.4. Data Analysis

Data were analyzed using the computer program SPSS 22 (SPSS Inc., Chicago, IL, USA). Analyses involved descriptive as well inferential statistics. Descriptive analysis was conducted based on frequencies and percentages performed for categorical variables. A Chi-squared test was used to assess the associations between some variables. Logistic regression was used to calculate some odds ratios (OR) with 95% to identify factors affecting contraceptive use. A *p* value less than 0.05 was used to indicate statistical significance.

### 2.5. Ethics Approval and Consent to Participate

All the participants read, understood, and signed the written consent form prepared by the study team. The study protocol was approved by the Jazan University Ethical Committee [REC38/8-S026]. Participants were told at the beginning that they could choose to participate in or to withdraw from the study at any time.

## 3. Results

The survey included 93.7% of the target number of women (450 out 480). Table 1 illustrates their background characteristics, as well as their use of and desire to use contraceptive methods. The majority (66.7%) of them were well educated, and 35.3% were working. The table reveals that 71.4% of participants were married, while 37.3% of the women had 1–2 children. The prevalence of ever having used any method of contraception was 64.9%, with a 95% CI [60.4–69.2]. The prevalence differed significantly by age group, job status, number of children ever born, and the number of pregnancies (*p* < 0.05 for all). The prevalence of the desire to use contraceptives in the future was high, at 70.9%, with a 95% CI [66.5–74.9]. It differed significantly by age group and job status (*p* < 0.05 for both).

**Figure 1:**
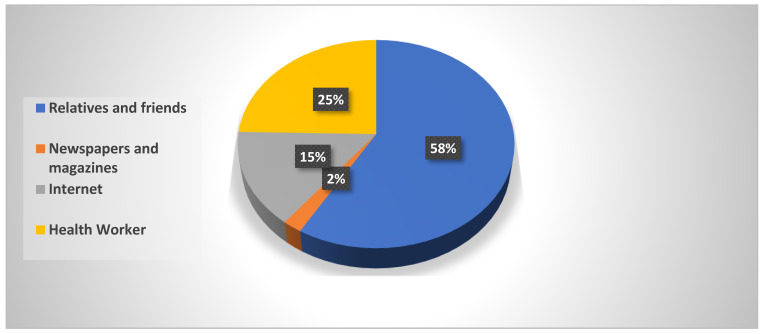
Women’s responses regarding sources of modern contraceptives.

Table 2 shows that almost 95.6% of participants had heard about a contraceptive method. Around 75% of them were familiar with pills. About 70.7% of the women thought the best place to find contraceptives was at a pharmacy. When asked about side effects, 32.3% of participants indicated they knew about hair loss, 35.4% about nervousness, and only 16.6% about weight gain. The ways they had heard about contraceptive methods differed by age group (*p* < 0.05). As Figure 1 reveals, the main sources of information about modern contraceptives were relatives and friends (58%), health workers (24%), and the internet (15%).

**Figure 2:**
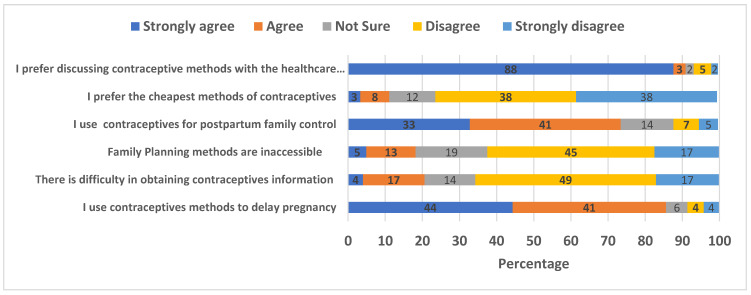
Women’s attitudes toward modern contraceptives.

Of the women in the sample, 85% had positive attitudes toward the use of contraceptive methods to delay pregnancy. A substantial number (66%) disagreed about the difficulty in obtaining information about contraceptive methods from healthcare professionals, while 62% disagreed about the inaccessibility of family planning services. The majority (74%) had positive attitudes toward postpartum use (Figure 2).

Table 3 displays the pattern of contraceptive use among study participants according to age group. About one third (36.2%) of women used pills as the most common type. Most women (32.9%) had used contraceptives for 2–4 years. The highest percentage of women (53.8%) cited child spacing as their reason for contraceptive use, followed by improving child health (21.8%), and avoiding unwanted pregnancy (18.0%). There was a significant difference by age group in women’s responses to the factors presented in the table (*p* < 0.05 for all).

Univariate logistic regression analysis was conducted with “ever having used contraceptives” as the dependent variable and a set of predictors that was found to be significantly associated in the previous analysis (Table 4). The analysis revealed that the use of contraception among women was associated with the 20–35 age group (COR  =  7.7, 95% CI  =  4.4–13.5, *p* < 0.001) more than the 15–24 age group. Having more than one pregnancy and having more than one child were also associated with increased use of contraceptive methods (*p* < 0.001 for both).

## 4. Discussion

This research tried to assess the knowledge about, attitudes toward, and practices of contraceptive use among women in Jazan, Saudi Arabia through a cross-sectional epidemiologic study. The findings of the study seem to suggest that knowledge of modern family planning methods is very high among study participants, which is in accordance with most Saudi Arabian family planning literature [12,17]. Unfortunately, this high level of contraceptive knowledge is not translated into actual use, as the overall use was found to be 64.4%. Although the Saudi community witnessed a considerable socioeconomic change in women’s education and employment, which resulted in some fertility decline, the predominant culture encourages a high level of fertility.

Our results show that the use of contraceptives at any point was 64.4% among women in Jazan. This estimate is consistent with many studies conducted in Saudi Arabia, including Al-Husain et al., 2018 in Jeddah (69.7%) [16], and Albezrah, 2015 in Taif (67.7%) [17]. Another group of studies in Saudi Arabia found lower use rates than this, as in Kharif et al., 2017 [18] and Al Sheeha, 2010 (44.8%) [12]. Our estimate is also far less than what was provided by Alenezi and Haridi, 2021, who cited the percentage of women in northern Saudi Arabia who had ever used contraceptives as 85% [19]. When we compare our estimate of contraceptive use with those in other Arab countries, it is higher than those in most Middle Eastern countries. According to the UN Fertility and Family Planning Report [20] 2020, the rates of contraceptive use are 43.2% in Egypt, 31.1% in Jordan, 19.6% in the Sultanate of Oman, 35.1% in Iraq, 31.6% in Syria, 32.2% in Bahrain, 35.5% in Kuwait, and 29.1% in Qatar. This variation may be attributed to various factors related to religious, cultural, social, and population policies in different countries.

The present analysis revealed that the highest percentages of contraceptive users were housewives (80.9%) and employed women (76.7%). This pattern is similar to that found in a study in the Aljouf region in the KSA [21]. Female students’ contraceptive use was the lowest among the study participants. The main reason was that those students still had a desire for more children, as they were mainly in young couples. It is well known that the age at first marriage is low in South Saudi Arabia. Our results revealed that the main source of information for participants was relatives and friends (58%). This finding is similar to those of other studies conducted in the KSA that also reported relatives and friends as being the main source of information [22,23]. A regional study conducted in Qatar showed that the most cited source of knowledge was friends (80%) [24].

The present analysis revealed that women were most familiar with oral contraceptive pills (74.9%) and intrauterine devices (69.9%). This is, to some extent, similar to the findings of previous studies conducted in Saudi Arabia and most Arab countries [12,21,22].

An analysis of factors that encouraged participants to choose a particular contraceptive method showed that those with fewer side effects were preferred by 42.6% of women. A further 17.7% of participants chose methods recommended by family and friends. Notably, 15.6% stated that contraceptive use is a husband’s choice, which raises the issue of husbands’ involvement in and support of family planning. Engaging men can lead to better gender-equitable attitudes, which support contraceptive use [25].

The results revealed that the main reason for using contraceptives was child spacing (53.8%). This is consistent with many studies in KSA [19,26]. Most inhabitants of the Middle East are Muslim, and they use contraceptives for child spacing rather than for limiting birth. The predominant culture encourages fertility and reproduction, and new couples are always under pressure to have their first baby. The results of the logistic regression model showed that the use of contraception among women was associated with age increase. Having more than one pregnancy or more than one child was also associated with the increased use of contraceptive methods. This result is consistent with those of Kharif et al., 2017 [18], who concluded that the number of children is a positive predictor of contraceptive use, and the increase in the number of children is associated with the likelihood of contraceptive use among the study participants.

Finally, according to the recent Saudi Household Health Survey conducted in 2018, the total fertility rate (TFR) average number of children born to a woman over her lifetime for Jazan was 1.8 live births [27]. This measure may be associated with contraceptive use among women in the Jazan region. Our results showed that two thirds of the women used contraceptives. Liu and Raftery (2020) documented the role of contraceptives and education in accelerating fertility decline [28]. More research is needed to investigate the association between contraceptive use and fertility decline in Saudi Arabia, especially in the Jazan region, which recorded below replacement fertility [27].

This research has some limitations. First, there is the cross-sectional nature of the study design, due to which the relationships between variables should be described as general associations and not causal relationships. Second, the study involved only one city, so the outcome could not be generalized to the whole Jazan region. Despite these limitations, this research offers an in-depth analysis of the family planning profile in southwest Saudi Arabia. The findings from this study will provide updated information about women’s knowledge, attitudes, and practices regarding family planning, and will help policymakers to develop appropriate interventions to improve women’s decisions in that respect.

## 5. Conclusions

The study showed that there is an increased awareness of contraceptive use among Saudi women in Jazan. Relatives and friends were the most common source of knowledge, while the most used methods were pills and IUDs. Women were using contraceptives to maintain gaps between pregnancies. More efforts are needed to improve women’s awareness of family planning options to ensure better utilization of the available services. Health education programs and counseling regarding family planning in health care settings are necessary for increasing the knowledge and use of contraceptive methods. Contraception awareness programs should be extended to men to ensure better utilization of the available family planning services.

## Figures and Tables

**Table 1:** Background characteristics, use and desire to use contraceptives among the study participants (*n* = 450).

Characteristic		All Women		Ever Used			Desire to Use		
		*n*	%	*n*	%	*p* Value	*n*	%	*p* Value
Age (years)	15–19	34	(7.6)	9	(26.5)	<0.001	12	(35.3)	<0.001
	20–24	121	(26.9)	51	(42.1)		84	(69.4)	
	25–29	68	(15.1)	47	(69.1)		57	(83.8)	
	30–34	98	(21.8)	78	(79.6)		78	(79.6)	
	35–39	59	(13.1)	48	(81.4)		41	(69.5)	
	40–44	54	(12.0)	45	(83.3)		36	(66.7)	
	45–49	16	(3.6)	14	(87.5)		11	(68.8)	
Educational Level	Illiterate	10	(2.2)	7	(70.0)	0.614	5	(50.0)	0.332
	Educated	140	(31.1)	95	(67.9)		99	(70.7)	
	Well educated *	300	(66.7)	190	(63.3)		215	(71.7)	
Job Status	Housewife	152	(33.8)	123	(80.9)	<0.001	116	(76.3)	0.028
	Student	139	(30.9)	47	(33.8)		87	(62.6)	
	Worker	159	(35.3)	122	(76.7)		116	(73.0)	
Children Ever Born	None	54	(15.3)	13	(24.1)	<0.001	38	(70.4)	0.213
	1–2 children	132	(37.3)	109	(82.6)		101	(76.5)	
	3–4 children	102	(28.8)	94	(92.2)		86	(84.3)	
	5 and more	66	(18.6)	59	(89.4)		50	(75.8)	
Pregnancies	None	45	(12.6)	6	(13.3)	<0.001	33	(73.3)	0.767
	1–2	122	(34.3)	99	(81.1)		95	(77.9)	
	3–4	99	(27.8)	89	(89.9)		80	(80.8)	
	5 and more	90	(25.3)	81	(90.0)		69	(76.7)	
All women		450	(100)	292	((64.9)		319	(70.9)	
95% CI				(60.4–69.2)			(66.5–74.9)		

* The well-educated category includes women who had undertaken undergraduate and postgraduate studies.

**Table 2:** Women’s awareness of contraceptive methods by age group (*n* = 450).

Characteristics		Proportion Who Answered Yes								*p* Value
		All Women		Age Groups (Years)						
				15–24		25–34		35–49		
		*n*	%	*n*	%	*n*	%	*n*	%	
Heard about contraceptive methods	Any method	432	(96.2)	139	(90.3)	165	(99.4)	128	(99.2)	<0.001
	Heard about calendar (date)	119	(26.4)	38	(24.5)	48	(28.9)	33	(25.6)	0.648
	Heard about oral contraceptive pills	338	(75.1)	115	(74.2)	121	(72.9)	102	(79.1)	0.452
	Heard about intrauterine devices	314	(69.8)	105	(67.7)	117	(70.5)	92	(71.3)	0.783
	Heard about injection methods	171	(38.0)	57	(36.8)	62	(37.3)	52	(40.3)	0.810
	Heard about condoms	155	(34.4)	61	(39.4)	56	(33.7)	38	(29.5)	0.211
Knowledge about sources	PHCCs	124	(27.6)	48	(31.0)	43	(25.9)	33	(25.6)	0.501
	Hospitals	229	(50.9)	88	(56.8)	84	(50.6)	57	(44.2)	0.107
	Pharmacies	319	(70.9)	114	(73.5)	119	(71.7)	86	(66.7)	0.428
Aware of contraceptive side effects	Hair loss	132	(32.3)	31	(24.4)	57	(36.3)	44	(35.2)	0.072
	Nervousness	144	(35.4)	33	(26.4)	60	(38.2)	51	(40.8)	0.037
	Weight gain	68	(16.6)	16	(12.6)	34	(21.5)	18	(14.4)	0.097

**Table 3:** Pattern of contraceptives use among study participants by age group (*n* = 450).

Factors		All Women		Age Groups (Years)						*p* Value
				15–24		25–34		35–49		
		*n*	%	*n*	%	*n*	%	*n*	%	
Current use of contraceptive methods	Calendar (date)	20	(4.4)	4	(2.6)	9	(5.4)	7	(5.4)	<0.001
	Oral contraceptive pills	163	(36.2)	32	(20.6)	66	(39.8)	65	(50.4)	
	Intrauterine device	110	(24.4)	38	(24.5)	40	(24.1)	32	(24.8)	
	Contraceptive injection	5	(1.1)	0	(.0)	3	(1.8)	2	(1.6)	
	Condoms	5	(1.1)	1	(.6)	4	(2.4)	0	(.0)	
	Withdrawal	6	(1.3)	3	(1.9)	2	(1.2)	1	(.8)	
	Other methods	74	(16.4)	42	(27.1)	28	(16.9)	4	(3.1)	
	Not using	67	(14.9)	35	(22.6)	14	(8.4)	18	(14.0)	
Duration of contraceptive use	Less than a year	75	(16.7)	22	(14.2)	32	(19.3)	21	(16.3)	<0.001 #
	1–4 years	148	(32.9)	28	(18.1)	73	(44.0)	47	(36.4)	
	5 and more	60	(13.3)	6	(3.9)	20	(12.0)	34	(26.4)	
	Not applicable	167	(37.1)	99	(63.9)	41	(24.7)	27	(20.9)	
Reasons for contraceptive use	To improve child health	98	(21.8)	21	(13.5)	43	(25.9)	34	(26.4)	<0.001
	For child spacing	242	(53.8)	48	(31.0)	106	(63.9)	88	(68.2)	<0.001
	To avoid unwanted pregnancy	81	(18.0)	24	(15.5)	35	(21.2)	22	(17.1)	<0.001
	For socioeconomic reasons	44	(9.8)	14	(9.0)	20	(12.0)	10	(7.8)	<0.001
	Advised by health care worker	18	(4.0)	1	(.6)	13	(7.8)	4	(3.1)	<0.001
What factors encouraged you to use this method	Offered for free	14	(3.1)	4	(2.6)	6	(3.6)	4	(3.1)	<0.001 #
	Fewer side effects	192	(42.7)	37	(23.9)	92	(55.4)	63	(48.8)	<0.001
	Advertised on social media	28	(6.2)	3	(1.9)	16	(9.6)	9	(7.0)	<0.001
	Husband’s choice	70	(15.6)	14	(9.0)	37	(22.3)	19	(14.7)	<0.001
	Family and friends	80	(17.8)	22	(14.2)	33	(19.9)	25	(19.4)	<0.001

# *p* value is based on Fisher’s exact test.

**Table 4:** Univariate logistic regression model for factors associated with ever having used contraceptives.

Variables	*p* Value	COR	95% CI for OR	
			Lower	Upper
Age group in years				
Less than 25	REF			
20–35 years	<0.001	7.7	4.4	13.5
35–49 years	0.114	1.6	0.9	2.8
Occupation				
Housewife	<0.001	8.3	4.9	14.2
Student	0.367	1.3	0.7	2.2
Worker	REF			
Children ever born				
None	REF			
1–2 children	<0.001	14.9	6.9	32.2
3–4 children	<0.001	37.1	14.2	96.2
5 and more	<0.001	26.6	9.7	72
Pregnancies				
None	REF			
1–2	<0.001	28.0	10.5	73
3–4	<0.001	57.9	19.6	170
5 and more	<0.001	58.5	19.4	175

Abbreviations: REF = references; COR = crude odds ratio; CI = confidence interval.

## Data Availability

This study has no additional supporting data to share.
